# Placental Growth Factor Contributes to Micro-Vascular Abnormalization and Blood-Retinal Barrier Breakdown in Diabetic Retinopathy

**DOI:** 10.1371/journal.pone.0017462

**Published:** 2011-03-07

**Authors:** Laura Kowalczuk, Elodie Touchard, Samy Omri, Laurent Jonet, Christophe Klein, Fatemeh Valamanes, Marianne Berdugo, Pascal Bigey, Pascale Massin, Jean-Claude Jeanny, Francine Behar-Cohen

**Affiliations:** 1 Institut National pour la Santé Et la Recherche Médicale (INSERM) U872, Physiopathology of ocular diseases: Therapeutic innovations, Paris, France; 2 Centre de Recherche des Cordeliers, Université Pierre et Marie Curie (UPMC), UMR S 872, Paris, France; 3 Université Paris Descartes, UMR S 872, Paris, France; 4 Fondation A. De Rothschild, Paris, France; 5 INSERM U640, Paris, France; 6 Centre National de la Recherche Scientifique (CNRS) UMR8151, Paris, France; 7 Université Paris-Descartes, Chemical and Genetic Pharmacology Laboratory, Faculté de Pharmacie, Paris, France; 8 Ecole National Supérieur Chimie Paris (ENSCP) Chimie ParisTech, Paris, France; 9 Assistance Publique – Hôpitaux de Paris (AP-HP) Université Paris 7, Hôpital Lariboisière, Department of Ophthalmology, Paris, France; 10 AP-HP Hôpital Hôtel-Dieu, Paris, France; German Heart Center, Germany

## Abstract

**Objective:**

There are controversies regarding the pro-angiogenic activity of placental growth factor (PGF) in diabetic retinopathy (DR). For a better understanding of its role on the retina, we have evaluated the effect of a sustained PGF over-expression in rat ocular media, using ciliary muscle electrotransfer (ET) of a plasmid encoding rat PGF-1 (pVAX2-rPGF-1).

**Materials and Methods:**

pVAX2-rPGF-1 ET in the ciliary muscle (200 V/cm) was achieved in non diabetic and diabetic rat eyes. Control eyes received saline or naked plasmid ET. Clinical follow up was carried out over three months using slit lamp examination and fluorescein angiography. After the control of rPGF-1 expression, PGF-induced effects on retinal vasculature and on the blood-external barrier were evaluated respectively by lectin and occludin staining on flat-mounts. Ocular structures were visualized through histological analysis.

**Results:**

After fifteen days of rPGF-1 over-expression in normal eyes, tortuous and dilated capillaries were observed. At one month, microaneurysms and moderate vascular sprouts were detected in mid retinal periphery *in vivo* and on retinal flat-mounts. At later stages, retinal pigmented epithelial cells demonstrated morphological abnormalities and junction ruptures. In diabetic retinas, PGF expression rose between 2 and 5 months, and, one month after ET, rPGF-1 over-expression induced glial activation and proliferation.

**Conclusion:**

This is the first demonstration that sustained intraocular PGF production induces vascular and retinal changes similar to those observed in the early stages of diabetic retinopathy. PGF and its receptor Flt-1 may therefore be looked upon as a potential regulatory target at this stage of the disease.

## Introduction

Pathogenic events in diabetic retinopathy (DR) include capillary basement membrane thickening, loss of microvascular intramural pericytes and leaky dilation [1]–[2]. At more advanced stages, capillary occlusion induces retinal ischemia and subsequent retinovitreal neovascularization causing bleeding and traction retinal detachments. Since its discovery in ocular fluids from patients with proliferative diabetic retinopathy (PDR) [3]–[4], VEGF has been recognized as a major pro angiogenic factor produced in response to hyperglycaemia and ischemia. Even though few animal models reproduce one or more of the DR lesions with unquestionable validity, non-diabetic animal models have been exploited to study retinal neovascularization pathogenesis [5]. The retinopathy of prematurity model is commonly used to study ischemic-related retinal diseases [6]–[7]. The angiogenic effects of VEGF have also been analyzed in transgenic mice models of ocular neovascularizations [8]–[9] or using viral VEGF gene transfer [10]–[11]. Whilst VEGF blockade has been demonstrated to efficiently reduce neovascularization progression and leakage in age-related macular degeneration, its exact place in the management of DR remains to be clarified. Intravitreal anti-VEGFs do improve vitrectomy outcome in cases of severe PDR [12] but should be administered shortly before surgery to avoid traction retinal detachments [13]–[14], and may potentially aggravate retinal ischemia. The effectiveness of anti VEGF therapies in patients with diabetic macular edema remains disputable [15] and suggests that other hypoxia-induced molecular factors may be involved such as PDGF, IGF-1, HGF, bFGF/FGF-2 [16]. Among them, placental growth factor (PGF) found at high levels in the vitreous [17]–[19] and the retina [20] of diabetic patients may have particular interest.

PGF is a homologue of VEGF [21] which binds to Flt-1 and to the co-receptors neuropilin−1 and −2, but does not bind to Flk-1 [22]. During vascular development, PGF is redundant with VEGF but, in pathological conditions, it acts directly on Flt-1-mediated endothelial migration and vascular permeabilization, and indirectly on Flk-1-mediated angiogenesis [23]. Whilst PGF chemotactic and angiogenic properties were highlighted early in the rabbit cornea [24], its implication in angiogenic diseases has been controversial. When over-expressed in VEGF-producing cells, VEGF/PGF heterodimers induced either inhibition [25]–[26] or enhancement [22], [27] of the VEGF pro angiogenic effects by interfering with Flk-1 binding. Similarly, the effect of hypoxia on PGF expression appeared to be cell-dependent [27]–[31]. In the retina, all the members of the VEGF family are expressed, primarily in ganglion cells, and up-regulated during ischemia. Moreover, we have previously shown that, under hypoxic conditions, RPE cells express PGF-1 and its receptor Flt-1 [19].

Over the last decade, the direct or indirect pro angiogenic effect of PGF was demonstrated during ischemia, inflammation, wound healing and cancer [32]–[34], as well as in skin angiogenesis [35]. In the eye, loss of PGF does not hamper retinal development [36] but impairs choroidal neovascularization [37], as does the loss of Flt-1 [38]. Intravitreal injections of PGF prevent oxygen-induced retinal ischemia, without inducing neovascularization [39]. Acute injection of PGF in the rat vitreous also altered the outer retinal barrier [19]. Taken together, these results tend to show that PGF may be involved in retinal pathology, but the effects of a sustained over-expression of PGF have not been studied.

We have recently developed a new non-viral method for gene transfer, based on electrotransfer (ET), using the ciliary muscle as a local bio-factory for the production secreted proteins [40]–[44]. In the present study, we used this method to characterize clinical and histo-pathological ocular changes induced by sustained secretion of rat PGF-1 (rPGF-1) in the rat eye *in vivo*.

## Methods

### Animals and fibrovascular membranes

Investigations were performed in accordance of the ARVO statement for the Use of Animals in Ophthalmic Vision Research. The study was approved by the Regional Ethics Committee in Animal Experiment N°3 of Ile-de-France region (approval p3/2008/062). Eighteen non-dystrophic Royal College of Surgeon's (cRCS: RCS- rdy+/p−) rats aged two months, from our facility, were first used as non pigmented and non-diabetic model to follow up the effects of PGF over expression. Due to the difficulty to observe albino eye fundi by fluorescein angiography (FA), Brown-Norway (BN) rats were then chosen as pigmented model, and purchased from Charles-River (L'Arbresle, France; N = 15) or from Janvier (Le Genest Saint Isle, France; N = 5). Eleven Goto-Kakizaki (GK) male rats, a model of non obese type 2 diabetes and three non-diabetic GK female rats from our facility were used to study PGF expression in diabetic conditions. All experiments were performed under ketamine/chlorpromazine (80 mg/kg/0.5 mg/kg) anesthesia. During clinical follow up, 1% tropicamide was used to dilate pupils. Animals were sacrificed by carbon dioxide inhalation.

Two fibrovascular pre-retinal membranes were obtained from eyes with PDR during closed microsurgery at the Lariboisière Hospital. Membranes were kept at −80°C immediately following removal, until Tissue-Tek® OCT (Sakura Finetek Europe, Zoeterwoude, Netherlands) embedding.

### Plasmids and ciliary muscle electrotransfer

The pVAX2-rPGF-1 plasmid is a 3.4 kb vector encoding rPGF-1 which was constructed after isolation, reverse transcription and amplification of rPGF-1 mRNA from rat placenta in order to limit the effect of immune response. After injection of 30 µg of plasmid (in 10 µL in saline) in the ciliary muscle using the trans-scleral route, electrotransfer was performed as previously described (40–43). Control eyes received ET of saline or ET of naked pVAX2 plasmid. Because no significant differences were observed between the groups of rats treated with ET of either saline or the naked plasmid, we have only represented the group of rats treated with ET of saline for more clarity in the different graphs.

### Clinical follow up and experimental design

Non-pigmented rats were firstly separated into two groups: a control groups (saline ET, N = 6, pVAX2 ET, N = 6) and a “treated” group (pVAX2-rPGF-1 ET, N = 6). The eye fundi were observed by slit lamp examination at days 4, 14 and 30 after ET. At this time point, one half of the rats were sacrificed for further analysis, and the other half was kept until day 100 for histological analysis. Controls (N = 10) and treated (N = 5) pigmented BN rats were examined by FA at weeks 4 and 6 after ET. For this purpose, fluorescein (0.2 mL of 10% fluorescein in saline) was administrated intraperitoneally. Angiograms were established using the Pro-3 Fundus Camera (Kowa) and saved on the Lhediaph LK3 software (L'Heritier). At two months, rPGF-1 expression was controlled by RT PCR and immuno-histochemical analyses were performed. Five more BN rats (N = 3 treated + 2 control) were follow up, between weeks 2 and 5 after ET, with a confocal scanning laser ophthalmoscope (cSLO) (HRA, Heidelberg Engineering, Dossenheim, Germany) [45]. The following grading system was used to quantify vascular changes as observed on angiography: Grade 0, normal retinal vasculature; +1 point for each of the following changes: dilated or tortuous vessels, microaneurysmal-like hyperfluorescent dots <10 or hyperfluorescence around the optic nerve head; +2 points for microaneurysmal-like hyperfluorescent dots >10.

Finally, GK rats (N = 4 treated + 4 control) were analyzed by immuno-histochemistry, one month after ET. Blood sugar level were measured with the Accu-Check active® system (Roche Diagnostics, Meylan, France).

### Polymerase Chain Reactions (PCR)

After dissection under an operating microscope, “ciliary muscle/bodies- iris” complexes and retinal tissues were removed and kept at −80°C until use. For each sample, total mRNA was isolated using the RNeasy plus mini kit (Qiagen, Courtaboeuf, France) following the manufacturer's instructions. One µg of total RNA was readjusted according to the RNA optic density at 260 nm, and then single-stranded cDNA was synthesized using random primer and superscript reverse transcriptase (Invitrogen, Cergy Pontoise, France).

#### Reverse Transcription PCR

cDNA from “Ciliary muscle/bodies- iris” complexes were amplified with rPGF-1 (sense, 5′-ATG CTG GCC ATG AAG CTG TTC-3′; antisense, 5′-ACT GAG GAA CCC CAC CTG TGA-3′), rat VEGF-A_165_ (sense, 5′-TTCATGGATGTCTATCAGCG-3′; antisens, 5′-GCTCATCTCTCCTATGTGCT-3′) and GAPDH (sense, 5′-ATG CCC CCA TGT TTG TGA TG-3′; antisense, 5′-ATG GCA TGG ACT GTG GTC AT-3′)-specific primers. These primers were designed to amplify cDNA fragments of 477 bp for rPGF-1, 234 bp for VEGF and 162 bp for GAPDH. For rPGF-1 and GFAP amplifications, thirty cycles of three PCR steps made of 1 minute at 94°C, 2 min at 56°C and 3 min at 72°C were performed. For VEGF amplification, after one step at 94°C, 2 min, forty cycles of 30 sec at 94°C, 1 min at 55°C and 1 min at 72°C were performed. PCR fragments were analyzed by 2.5% agarose gel electrophoresis and visualized by ethidium bromide staining under UV. The relative band intensities for both growth factors were calculated in comparison with that for GAPDH.

#### Real-Time PCR

For retinal extracts, reverse transcription was followed by real time PCR using 5 µl cDNA, TaqMan® Universal PCR Master Mix, No AmpErase® UNG for the following primers (Applied Biosystems CA, USA): 18S (Hs99999901_s1); TNF-alpha (Rn01525860_g1); IL-1beta (Rn00676333_g1); IL-6 (Rn01410330_m1); VEGF-A (Rn01511608_m1). The reactions were performed on 7300 Real-Time PCR System (Applied Biosystems CA, USA) in 40 cycles of 15 sec at 95°C, 1 min at 60°C. Product was not generated in control reactions in which cDNA were omitted during synthesis. Relative quantification of real-time PCR results was performed by using the “delta-delta Ct method”.

### Flat-mounts of neuroretinas and of the remaining RPE-choroid-sclera complexes

After fixation of whole eyes, 15 min in paraformaldehyde (PAF) 4% solution in saline, anterior segments were discarded and the neuroretinas were carefully separated. Post-fixation was carried out for 10 min at −20°C in methanol for retinas and in acetone for RPE-choroid-sclera complexes. After rehydratation with PBS Triton X 100 1% solution, neural retinas were incubated overnight with the FITC-conjugated lectin from *Bandeiraea simplicifolia* (1∶100; Sigma-Aldrich, St-Quentin Fallavier, France) and, RPE-choroid-sclera complexes, with a polyclonal rabbit anti-occludin antibody (1∶400; Invitrogen). Occludin was visualized by incubation with an Alexa-594-conjugated goat anti rabbit IgG (1∶200; Invitrogen). After washing, tissues were flat-mounted in gel mount (Biomeda Corp., Foster City, CA, USA) and examined with a fluorescence microscope Olympus BX51 (Rungis, France) coupled with a digital camera Olympus DP70, or with a confocal microscope Zeiss LSM 710 (Le Pecq, France).

### Histological analysis

Eyes were fixed in PAF 4% and glutaraldehyde 0.5% solution in saline for 2 hours, dehydrated and embedded in historesin (Leica Microsystems, Nussloch GmbH, Germany). Sections of 5 µm were stained with 0.5% toluidine blue (Serva, Paris, France) and observed with an Aristoplan microscope (Leitz) coupled with a Leica DFC480 camera.

### Immunohistochemistry

Dual immuno-staining were performed on 10-µm cryostat sections from non-pigmented eyes and diabetic human fibrovascular membranes, fixed 5 min in PAF 4% solution. The sections were incubated one hour at room temperature with a polyclonal goat antibody raised against rPGF-1 (1∶100, #sc-1883, Santa-Cruz Biotechnology, CA, USA), associated with a monoclonal mouse anti-alpha smooth muscle actin (alpha-SMA) antibody (1∶100; Chemicon International/*Millipore, Molshein, France*), or a polyclonal rabbit anti-glial fibrillary acidic protein (GFAP) antibody (1∶100; Dako Cytomation, Trappes, France), or a polyclonal rabbit von Willebrand factor (vW) antibody (1/100; Dako Cytomation). The secondary antibodies used (1∶250; Invitrogen) included an Alexa-488-conjugated rabbit anti-goat antibody, a Texas-Red-conjugated donkey anti-mouse antibody, and a donkey Alexa-594 conjugated anti-rabbit antibody. Controls involved the omission of the primary antibody. The sections were stained 5 min with 4′, 6 diamidino-2-phenylindole solution (DAPI, 1∶5000; Sigma-Aldrich, Saint-Quentin Fallavier, France), mounted in glycerol:PBS (1∶1), and viewed with the fluorescence microscope.

### Western-blot

As only male GK rats become diabetic with ageing, PGF immuno-blotting was performed on protein extracted from the entire retinas of three diabetic male rats - aged two, five and twelve months, and compared to PGF expression the neuroretina from three non diabetic GK female rats - aged one, five and twelve months. Neuroretinas were homogenized in lysis buffer (10 mM Tris-HCl PH 7.5, 1 mM EDTA, 1 mM EGTA, 150 mM NaCl, 0.5% Nonidet P40, 1% Triton X-100, β-mercaptoethanol) containing protease inhibitor cocktail (Roche). Forty µg of proteins were subjected to SDS-PAGE in a NuPage® 4–12% polyacrylamide gel (Invitrogen) and electroblotted onto a nitrocellulose membrane (Schleicher &Schuell BioScience, Dassel, Germany). The membrane was incubated with the anti-rPGF-1 antibody (1∶1000) over-night at 4°C, and then, incubated with a corresponding secondary antibodies, horseradish peroxidase-conjugated Fab fragment (1∶5000; Caltag, Burlingame, Canada), one hour at room temperature. Immuno-reactive bands were detected with the ECL Western Blotting Detection Reagents Kit (Amersham Biosciences, Orsay, France). To check for equal loading, membranes were probed with an anti-beta-tubulin antibody (Serotec, Düsseldorf, Germany).

### Statistics

PCR results and histological measurements were expressed as the mean±SEM. The Mann-Whitney U-test was used to determine differences between groups. A P value <0.05 was considered statistically significant.

## Results

### Effect the sustained over expression of r PGF-1 in non diabetic rat eye

**Figure 1:**
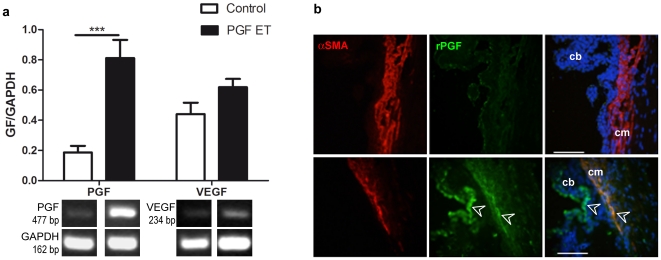
Control of rPGF-1 expression. **(a) PGF and VEGF expression analysis at the mRNA level, two months after pVAX2-rPGF ET.** Mean relative band intensity ±SEM determined after detection of rPGF-1, rVEGF and GAPDH mRNA expression in control eyes (in white, n = 5) or in pVAX2-rPGF-1 ET treated eyes (in black, n = 5); **, p = 0.0079. Representative RT-PCR products visualized by ethidium bromide staining are shown. **(b) Localization of rPGF-1 expression on sections of the ciliary muscle, one month after saline ET (Control) or pVAX2-rPGF ET (PGF ET).** Alpha-smooth muscle actin (αSMA) - Texas-Red labeling allows localization of the ciliary muscle (**cm**). Compared to control eye, white arrowheads indicate that after pVAX2-rPGF-1 ET, the protein of interest which is labeled in green, is localized in muscle fibers and in the epithelium of ciliary bodies (**cb**). No staining was observed when the primary antibody was omitted. Scale bars: 100 µm.

**rPGF over-expression was controlled at two months after ET.** PCR products from the circumferential ciliary region revealed an expected 477 bp rPGF-1 band in both the treated and control group, and showed that rPGF-1, but not VEGF, was significantly increased in pVAX2-rPGF-1 ET-treated eyes as compared to saline ET-treated eyes (p = 0.008) (Fig. 1a). Immuno-staining revealed rPGF-1 localization in the ciliary muscle and in the ciliary body epithelium (Fig. 1b).

#### The over-expression of rPGF-1-induced retinal vessels changes

***In vivo.*** At day 4, using indirect ophthalmoscopy on albinos rats, no difference between pVAX2-rPGF-1 ET-treated and control eyes were observed. At day 14 and 30, respectively one third (n = 4/12 eyes) and one half (n = 6/12) of the treated eyes presented tortuous vessels and microvascular abnormalities. At day 100, focal regions of sub retinal fluid accumulation were observed in half of the rPGF-1-treated eyes (n = 3/6), demonstrating progressive vessel abnormalization upon sustained PGF1 exposure.

**Figure 2:**
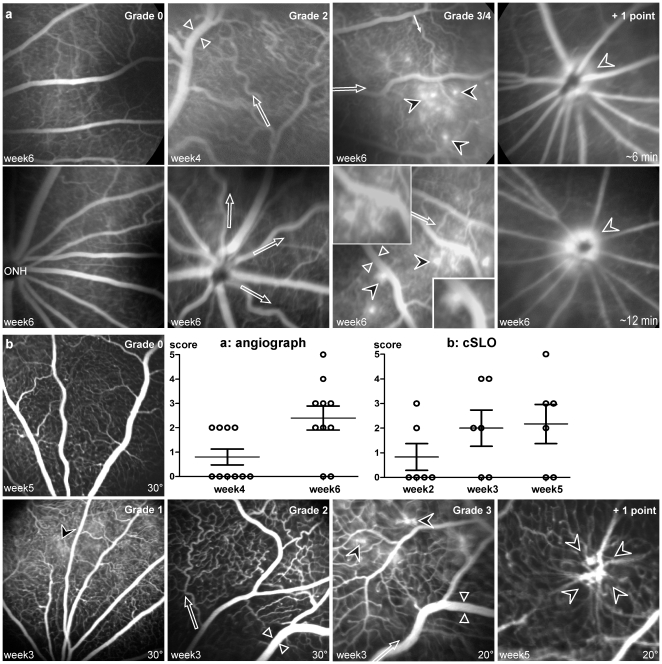
Fluorescein angiograms of Brown-Norway rat eyes. **(a) Observations with a classic angiograph (Pro III Fundus camera, Kowa), 4 and 6 weeks after pVAX2-rPGF ET**. Angiograms were established with a scan angle of 30°. Vascular abnormalities were scored from 0 to 5 in accordance to the following grading: **Grade 0**, normal retinal vasculature, as observed in control fundus at week 6 (**ONH**, Optic Nerve Head); **+1 point** for each of the following changes - dilated (between white arrowheads) or tortuous (white arrows) vessels, microaneurysmal-like hyperfluorescent dots (black arrowheads) <10 or hyper-fluorescence around the ONH; **+2 points** for microaneurysmal-like hyper-fluorescent dots >10. **(b) Observations with a confocal scanning Laser Ophthalmoscope (cSLO, Heidelberg Retina Angiograph I), 2, 3 and 5 weeks after pVAX2-rPGF ET**. The same grading was used to score vascular abnormalities. The higher resolution of the cSLO allowed the observation of early fluorescein leakage (**Grade 1**), and the detection of strong vascular abnormalities at later stage (**+ 1 point**).

Fluorescein angiography (FA) on pigmented eyes and the definition of a grading system (Fig. 2a) allowed a more precise analysis of the PGF-induced effects on retinal vasculature. Control rats showed normal vasculature throughout the follow-up period (Fig. 2a, Grade 0). Four weeks after ET, 40% of the PGF-treated eyes (n = 4/10) demonstrated dilated (mostly veins) and tortuous (mostly arterial) vessels, in central and peripheral retina (Fig. 2a, Grade 2). Six weeks after ET, 60% (n = 6/10) showed focal vessel leakage in the mid retinal periphery (Fig. 2a, Grade 3/4). Hyper fluorescent dots appeared as leaky micro-aneurysms. In three eyes, profuse dye leakage around the ONH increased with the angiographic sequence time, signing large vessel permeabilization (Fig. 2a, +1 point). No clear retinal neovascularization was observed at this time point.

The cSLO made it possible to carry out an accurate follow up of six more eyes in which rPGF-1 was overexpressed, compared to normal eyes (Fig. 2b, Grade 0). At week 3, their angiograms demonstrated vasodilation and vascular tortuosity (n = 4/6; Fig. 2b, Grade 2), and microaneurysms on capillaries (n = 2/6; Fig. 2b, Grade 3). At week 5, these four eyes presented more hyper-fluorescent dots on capillaries than earlier, along with focal neovascular sprouting (Fig. 2b, +1 point).

#### *Ex vivo*

**Figure 3:**
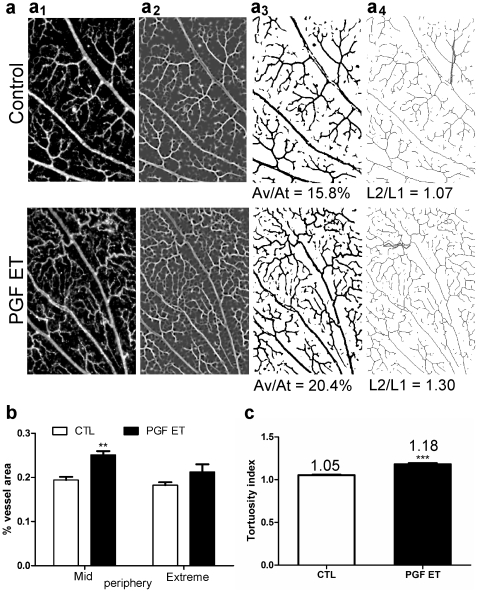
Morphological analysis of flat-mounted retina labeled with FITC-conjugated lectin from *Bandeira simplicifolia*. **(a) Illustration of the procedure followed with “ImageJ” software.** (**a_1_**) Selection of seven areas per group, in mid (700x500 px) and extreme (400x300 px) peripheries on mosaics (3007x2904 px) made with microscopic images at low magnification (x4). (**a_2_**) Application of steerable filters for ridge detection in the selected image, with the plug-in “Feature Detector” [57]. (**a_3_**) Transformation of the filtered image into a binary image allowing selection of the retinal vessels to calculate the vascularized area (area covered by vessels out of total area, **Av/At**). (**a_4_**) Derivation of the vascular skeleton from the binary image, using the procedure “skeletonizes”. This second binary picture was used to calculate the tortuosity index corresponding to the ratio: real vessel length (**L2**, in red) out of the length of an imaginary straight line on the measured vessel (**L1**, in blue) as shown in d_1_ and d_2_. **(b–c) Quantitative analysis of the retinal vasculature from control (CTL) and PGF ET-treated eyes. (b)** Vascularized area calculated from the binary images which are illustrated in c_1_ and c_2_. **All data**: CTL, 18.8%±0.5%; PGF-ET, 23.7%±1.1%; ** p = 0.0034. **Mid periphery**: CTL, 19.4%±0.7%; PGF-ET, treated eyes 25.1%±0.8%; Bonferroni, ** p<0.01. **Extreme periphery**: CTL, 18.2%±0.7%; PGF-ET treated eyes 21.2%±1.7%; Bonferroni, ns. (**c**) Tortuosity index of capillaries calculated from the second binary images. CTL, controls 1.053±0.005; PGF-ET, treated eyes 1.180±0.014; *** p<0.0001; PGF ET/CTL = 1.12.

Analysis of the flat-mounted retinas at low magnification (Fig. 3a) demonstrated that the lectin-labeled vascularized area was significantly increased in the rPGF-1 treated group as compared to the control group (23.7%±1.1% *vs* 18.8%±0.5%, p = 0.0034), particularly in the mid periphery (Fig. 3b), an area showing numerous tortuous capillaries (Fig. 3c).

**Figure 4:**
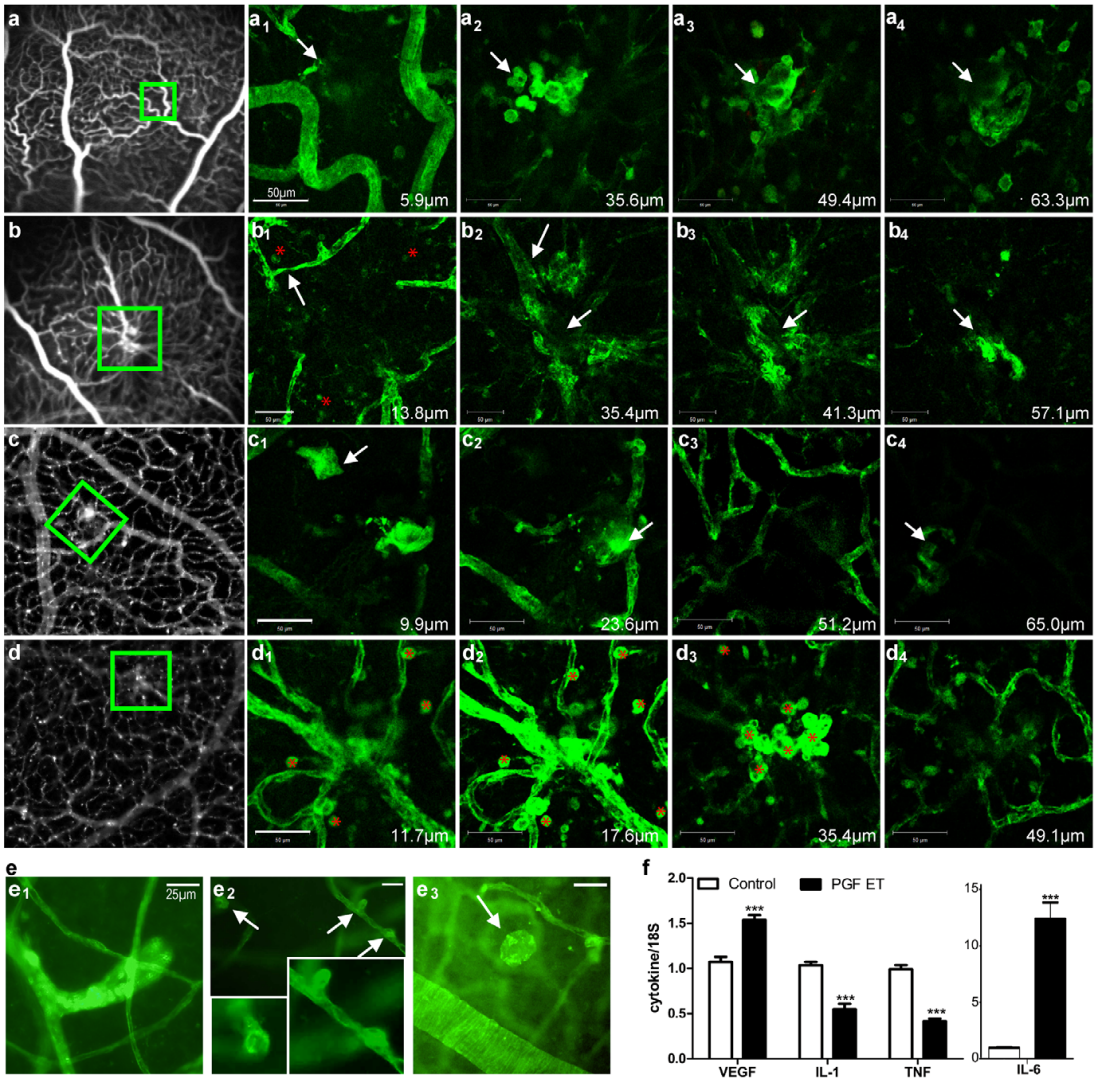
Angiogenic response on retinal vascular cells. **(a-d) Confocal microscopy of vascular abnormalities within the neural retina, one month after pVAX2-rPGF-1 ET.** Confocal microscopy of flat-mounted neuroretinas from eyes in which rPGF-1 was over-expressed shows lectin-positive blood vessels and cells at different depth levels, from the superficial (1) to the deep vascular plexus (4). Scale bars = 50 µm. (**a, b**) The abnormalities noticed *in vivo* on SLO angiograms from two eyes were found on retinal flat-mounts by comparison of the vascular architectures. (**c, d**) For illustration, retinal flat-mounts have been turned into grey-scale pictures. Red asterisks indicate the presence of infiltrating cells around vascular abnormalization. **(e) Optic microscopy of vascular abnormalities within flat-mounted retinas, two months after ET.** Vascular abnormalities (**e_1_**), sprouts (**e_2_**) and microaneurysmal-like structures (**e_3_**) were observed between the inner and the middle vascular beds. **(f) Q PCR analysis of VEGF, IL-1beta, TNF-alpha and IL-6, two months after ET,** in retina of control (in white, n = 3 in duplicate) and pVAX2-rPGF-1 ET treated (in black, n = 5 in duplicate) retina from BN rats. ***, p<0.005 *versus* wildtype mRNA expression. Data represent mean ± SEM.

Whereas some areas appeared unperfused on FA (Fig. 4a), magnified confocal imaging allowed to observe a strong superficial cellular infiltration leading to a vascular sprout lay in the lower capillary bed. Infiltrating cells were also observed around and in the superficial vicinity of the vascular knots appearing as hyper-fluorescent dots on FA (Fig. 4b). In the mid retinal periphery, others protrusions and vascular irregularities were identified (Fig. 4c–d). Two months after ET, intra-retinal vascular abnormalities (Fig. 4e): vascular loop, sprouting of endothelial cells and microaneurysms, were also observed.

At this time point, the expression of angiogenic and inflammatory mediators was evaluated at the mRNA level in the neuroretina (Fig. 4f). As compared to control eyes, rPGF-1 over-expression induced significant up regulation of IL-6 (more than 10 times increase) and VEGF, and down-regulation of IL-1beta and TNF-alpha.

#### rPGF-1-induced effects on retinal histology

**Figure 5:**
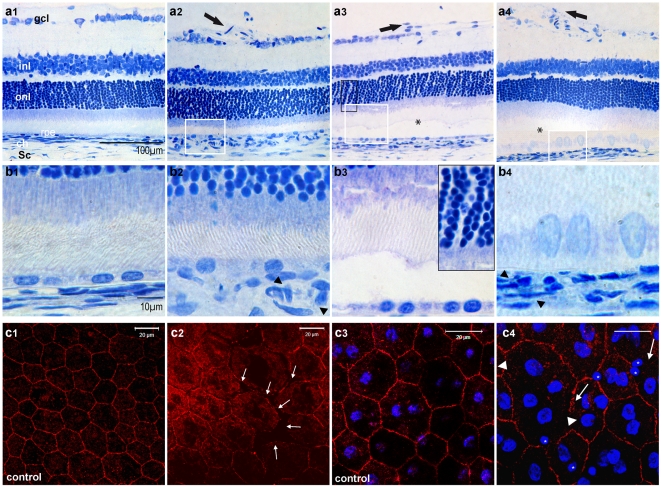
Effect of PGF over expression on retinal histology and on RPE cells. **(a–b) Histological sections of retinas from eyes embedded in historesin and stained by Toluidine blue, three months after saline (a_1_, b_1_) and pVAX2-rPGF-1 ET (a_2_–a_4_, b_2_–b_4_).** (**a_1_**) Normal histological section of the retina after pVAX2 ET. **ch**, choroid; **gcl**, ganglion cell layer; **inl**, inner nuclear layer; **onl**, outer nuclear layer; **rpe**, retinal pigmented epithelium; **Sc**, Sclera. Scale bar (a1–4)  = 100 µm. (**a_2_**) Histological section of pVAX2-rPGF-1 ET-treated retinas, showing vascular retinal abnormalities in the inner part of the peripheral retina. (**a_3_**) Sections showing retinal detachment (star) associated with pre-retinal proliferation (arrow) and edema at the ONL level (magnified in b3 inset). (**a_4_**) Sections showing retinal detachment (star) associated with RPE barrier breakdown. (**b_1_**) Section at high magnification showing normal RPE cells after PBS ET. Scale bar (a1–4)  = 10 µm. (**b_2_–b_4_**) Magnified RPE cells of the retinal sections (a_2_–a_4_) demonstrating morphological changes of RPE cells which appears swollen, and dilation of the chorio-capillaries (b_2_, b_4_ black arrowheads). Background has been subtracted in all pictures. **(c) Sustained blood-retinal barrier breakdown induced by rPGF-1 over expression.** Tight junctions were observed by occludin immuno-histochemistry on whole flat-mounted RPE cells. (**c1–3**) Normal tight junction-associated occludin was observed in PBS-ET treated eyes. Scale bar = 20 µm. (**c2–4**) Two months after pVAX2-rPGF-1 ET, junctions remained opened between some RPE cells (white arrows). At this opened junction level, infiltrating cells (stars) are also detected by the DAPI staining and recognized by the small size of their nuclei as compared to those of RPE cells.

Three months after pVAX2-rPGF-1 ET, historesin sections showed vascular abnormalities at the vitreoretinal interface, including focal vessel dilation (Fig. 5a_2_) and pre retinal vascular sprouting (Fig. 5a_3–4_). In the retina, focal detachments were observed in regions in which RPE cells were abnormal (Figure 5b_3_–b_4_), that is in favor of fluid accumulation in the sub retinal space. Changes in the morphology of RPE cells could be observed both on sections (Fig. 5b_4_) and on flat-mounted RPE (Fig. 5c_2_). These preparations also demonstrated focal disruptions of the tight-junctions, as shown by occludin staining, and the presence of infiltrating cells with small nuclei on the apical side of RPE cells (Fig. 5c_4_) which were not found in control eyes (Fig. 5c_3_).

### Endogenous expression of PGF in the time course of diabetic retinopathy in GK rats

Before over-expressing PGF in diabetic GK rats, we have evaluated whether the endogenous expression of PGF was modified in the retina of GK rats after different time of hyperglycemia.

**Figure 6:**
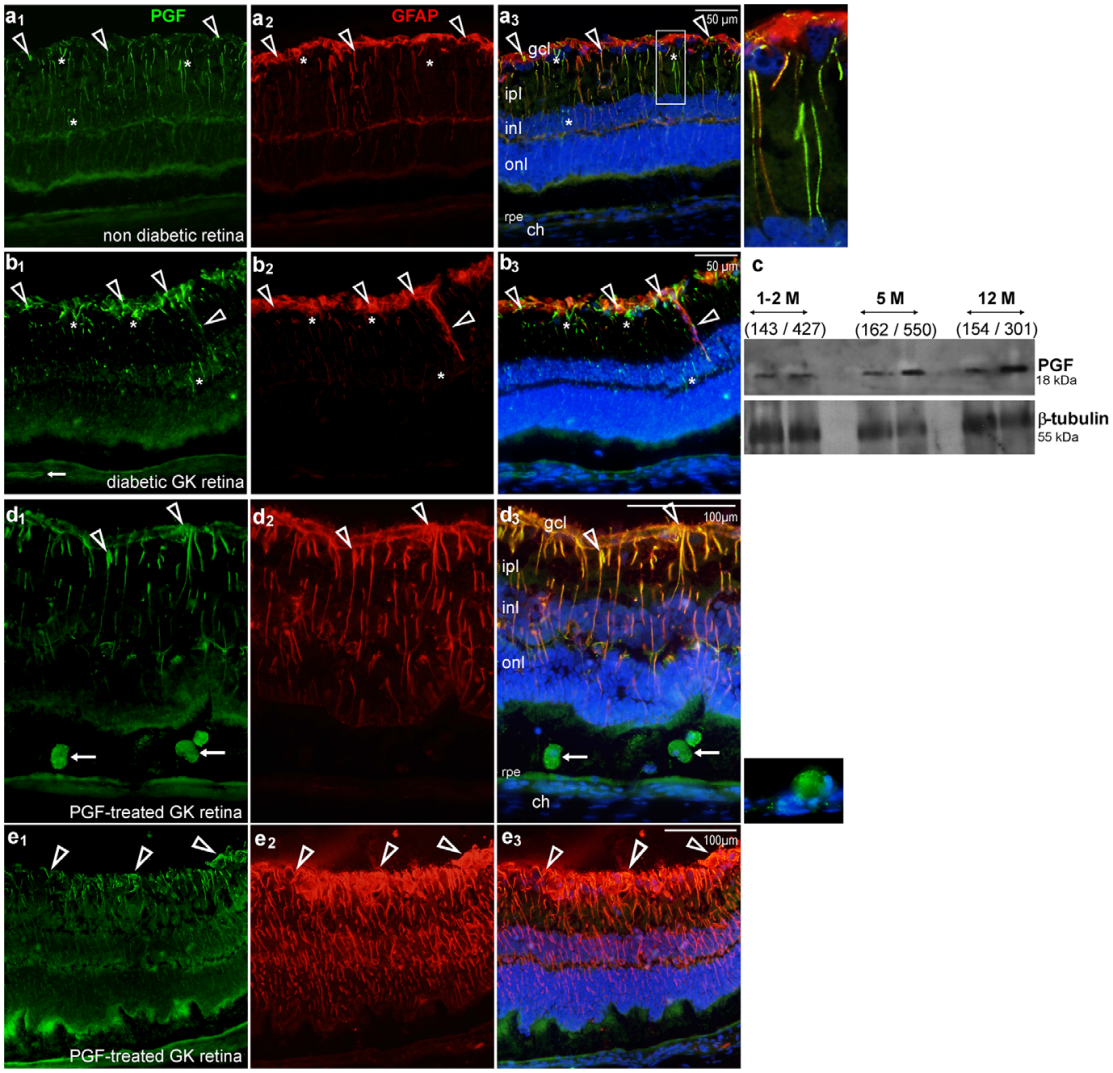
PGF expression in diabetic retina. **(a–b) Comparison of PGF staining in non diabetic (a) and diabetic (b) retinas.** (**a**) Sections of eyes from adult control non diabetic rat showed co-expression of PGF and GFAP in glial Muller cells from the gcl (arrowheads) to the inl, and PGF expression in glial Müller cells which are not immuno-reactive for GFAP (white star). Scale bar = 50 µm. (**b**) A similar pattern was observed on retinal sections of eyes from three-month-old diabetic rats, with a strong immuno-reactivity for PGF at the gcl level. **(c) PGF detection by Western-blot in diabetic and non-diabetic retinas, from 1, 2, 5 and 12 month-old rats**. For each lane in which 40 µg of proteins were deposited, the blood sugar level of the represented rats is indicated between parentheses. **(d–e) Immunostaining for PGF and GFAP in sections from pVAX2-rPGF-1 ET- treated diabetic rat eyes, one month after ET.** Sections show PGF-expressing infiltrating cells in the sub retinal space (**d**, arrows) and confirmed PGF expression by RMG cells (**d**, arrowheads). GFAP staining showed gliosis induced by RMG cells (**d, e**). **ch**, choroid; **gcl**, ganglion cell layer; **inl,** inner nuclear layer; **ipl**, inner plexiform layer; **onl**, outer nuclear layer; **rpe**, retinal pigmented epithelium; Scale bar = 100 µm.

In non-diabetic untreated eyes, PGF immuno-localization showed that PGF-1 is expressed in retinal Müller glial (RMG) cells and in the inner segments of photoreceptors (Fig. 6a). In three-month-old GK diabetic rats, PGF-1 was strongly expressed in the inner part the retina, mostly in astrocytes and in swollen RMG cells, as shown by the co labeling with the glial marker, GFAP (Fig. 6b). PGF expression over time in diabetic retinas was compared to PGF expression in control retinas using western-blot (Fig. 6c). This kinetic showed that PGF expression increased in the neuroretinas from diabetic rats aged between two and five months, and remained elevated as the diabetes develops at later stages (12 months), whereas it remained constant between one month and one year in retinas from non diabetic control rats. PGF-1 is therefore upregulated in the retina of GK diabetic rats, increasing at the early stages of diabetes and remaining elevated thereafter.

**Figure 7:**
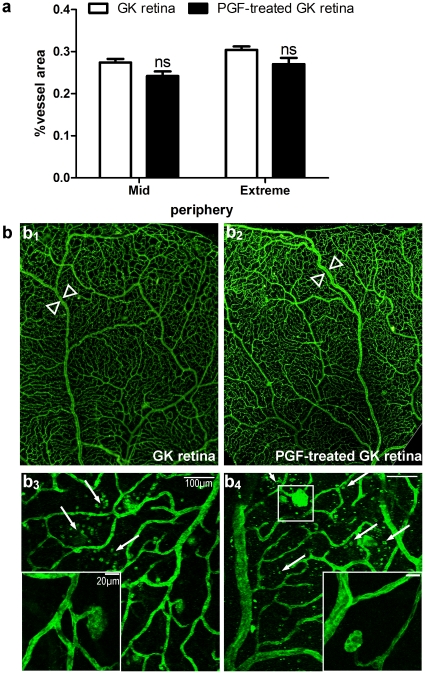
Analysis of flat-mounted diabetic retinas labeled with FITC-conjugated lectin from *Bandeira simplicifolia*, one month after ET. **(a) Quantitative analysis of the retinal vasculature from control and PGF ET-treated diabetic rat eyes. (b) Microscopy of flat-mounted control (b_1_, b_3_) and PGF ET-treated (b_2_, b_4_) diabetic retinas.** (**b_1_–b_2_**) Optic microscopy on mosaics made with microscopic images at low magnification (x4) showing no evident difference of the retinal vascularization between control (b_1_) and pVAX-2-rPGF-1 ET-treated eyes (b_2_), except a weak retinal venous dilation in treated retinas (between arrowheads). (**b_3_, b_4_**) Confocal microscopy showing lectin-labeled cell infiltration (arrow) and micro-aneurysmal-like structures observed in both group (insets).

In addition, analysis of the lectin-labeled flat-mounts, retinas from 5 months-old GK eyes demonstrated some vascular abnormalities. In normal GK retinas, vascularized area was higher (27.2%±0.03%) than in normal non diabetic retinas (18.8%±0.02%, p<0.0001%), and vessels appeared tortuous with some vascular irregularities (Fig. 7b_1_–b_3_).

### Effect of the sustained over expression of rPGF-1 in GK diabetic rat eyes

rPGF-1 over-expression in GK rats did not enhance or modify the vessel abnormalities observed in the control GK rats treated with either ET of saline or ET of naked plasmid. The vascularized areas were not statistically different in GK control rats and PGF-treated GK rats (Fig. 7a). Magnified observations highlighted cellular infiltration in diabetic retinas which seemed enhanced by rPGF-1 over expression (Fig. 7b_4_).

However, rPGF-1 induced intense glial activation. Indeed, strong GFAP staining was found in the retina from rPGF-1-treated eyes as compared to control diabetic retina and ET saline control diabetic retina (not shown). Indeed, we have previously shown that ET of ciliary muscle does not induce activation of inflammation markers because it does not imply perforation of the retina and the treatment area is anterior. rPGF-1 was mostly located in RMG cells, but it was also found in RPE cells and in large cells infiltrating the sub retinal space (Fig. 6d). Moreover, GFAP staining highlighted intense glial activation and aberrant glial proliferation at the retinal surface of rPGF-1-treated diabetic eyes as compared to untreated GK rats (Fig. 6e). As glial proliferation contributes to the early steps of peri-retinal membrane formation [46], PGF could intervene in the induction of pre-retinal gliosis observed in PDR. To check this hypothesis, we localized PGF by immuno-detection in two membranes excised from eyes with PDR.

### Immunohistochemical analysis of epiretinal membranes from diabetic patients

**Figure 8:**
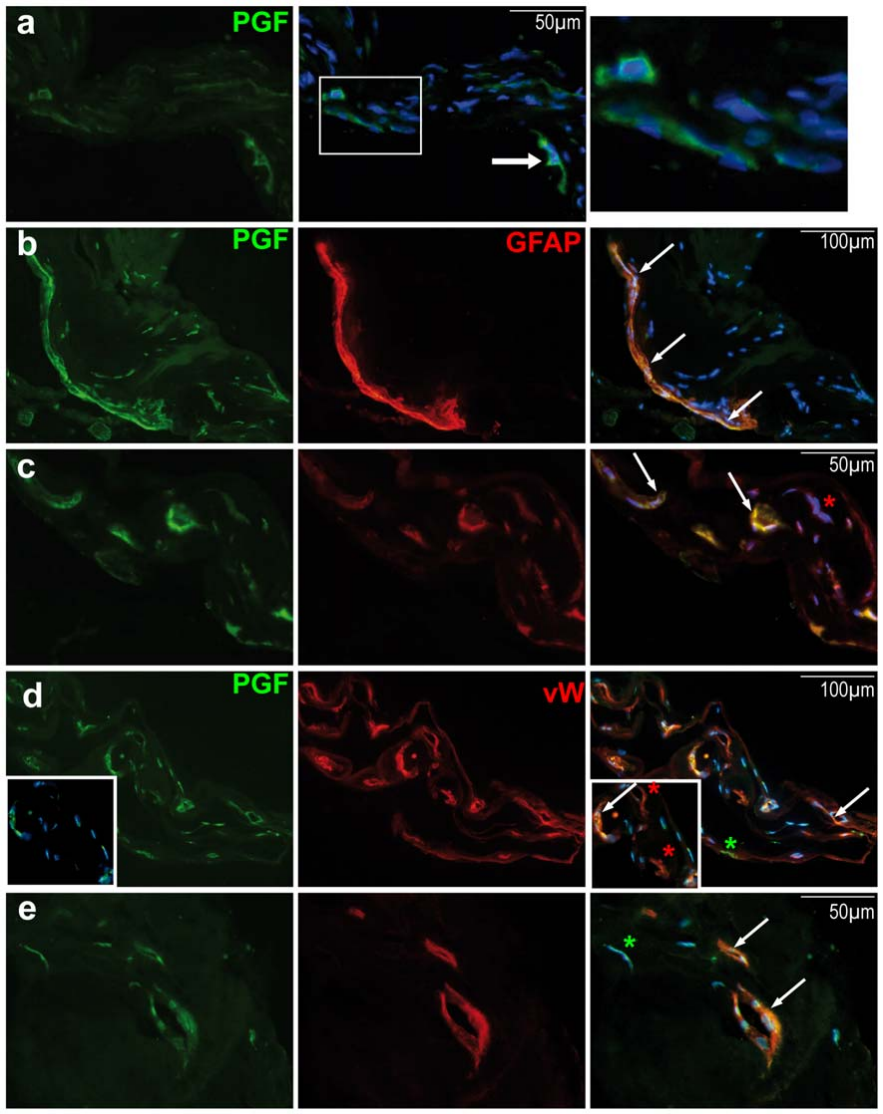
Immunostaining for PGF (a–e), GFAP (b, c) and von Willebrand factor (d, e) in membrane excised surgically from a 60-year-old woman with proliferative diabetic retinopathy. (**a**) PGF was immuno-detected on the epi-retinal membrane. It was localized in GFAP (**b–c**, arrows) and von Willebrand factor (vW) (**d–e**, arrows) immuno-positive cells. Red stars indicate cells expression only GFAP (c) or vW (d), and green stars show non-endothelial cells expressing PGF.

PGF was detected in peripheral cells of the pre-retinal membranes (Fig. 8a arrow) and in cells forming tubes, resembling vascular cells (Fig. 8a magnified). GFAP and von Willebrand factor co-staining demonstrated the fibro-vascular nature of the membranes (Fig. 8b–e). PGF was localized in some GFAP-positive (Fig. 8b–c) and in some vascular ECs (Fig. 8d–e).

## Discussion

The pro angiogenic effect of VEGF was demonstrated by the acute exposure of rat and monkey retinas, inducing an intense and quick pre retinal neovascularization [10]. Such pro angiogenic effects were not reported after acute exposure of the retina to PGF. Indeed, previous studies have shown that an acute administration of PGF in the mouse [39] or in the rat vitreous [19] had no effect on retinal vessels, suggesting that PGF had no clear pro angiogenic activity. However, in pathologic conditions, abnormalities are observed after years of sustained cytokinic and growth factors local deregulation. This is the reason why our model is based on a sustained secretion of PGF in the vitreous of normal or diabetic rat eyes.

The progressive retinal vessels abnormalization was followed up *in vivo* by FA. The release of rPGF-1 in ocular fluids had no effect after a week, but then chronologically induced the dilation and increased tortuosity of retinal vessels, the formation of microaneurysms and vascular sprouts together with increased vessel permeability. Nevertheless, no real pre-retinal neovessels have been observed over the experimental period. PGF effect therefore differs from VEGF which solely induces vitreous neovascularization. Interestingly, recent observations made in tumor vascularization pointed out that abrogation of PGF by different means did not influence vascular density but reduced significantly abnormalization of the vessels, such as tortuosity and disorganization [47]. Our results are in agreement with this observation, showing that PGF may be involved in the first phase of diabetic retinopathy when only microaneurysms are present. In GK rats, a model of type 2 diabetes, we showed that PGF progressively increased in the retina from 2 to 5 months and remained at high level for at least 12 months. Together with this endogenous PGF over-expression, we have observed vascular abnormalities, similar to those obtained in non diabetic rats by PGF over-expression. However, contrarily to humans, no pre-retinal neovascularization and no ischemic area are observed in this model, even at late stages. Interestingly, the over-expression of PGF in diabetic rats did not enhance the phenotype of diabetic retinopathy, even if it may have increased the cell infiltration. Other factors, among which the over expression of VEGF, are probably required to induce pre-retinal neo-vessels.

Taken together, these observations suggest that PGF may contribute to the early vascular abnormalization taking place in the early phases of DR. Under physiological conditions, the adult retinal vascular cells mostly express Flt-1 [48], and we have shown that in normal retina RMG cells express PGF, suggesting that PGF could intervene in the neurovascular control. PGF over-expressed in the neuroretina binds to its specific receptor, Flt-1, and contributes to alter the vessels wall leading to leakage, vessel dilatation and aneurysmal formations. Consistent with our finding, a recent study reported that VEGF and PGF ablate pericytes from the retinal vasculature through the ERK signaling pathway mediated by Flt-1, confirming its role in the breakdown of the inner blood retinal barrier [49]. The PGF-induced effects on retinal vasculature could result from a direct Flt-1 activation, but also from the PGF-induced VEGF displacement from Flt-1 and sFlt-1 toward Flk-1, which is over-expressed in pathological conditions. Moreover, hetero-dimerization of PGF with VEGF could exert mitogenic effects on endothelial cells by activating inter- and intra-molecular interactions between both VEGF receptors [23]. In addition, PGF indirectly stimulates angiogenesis by recruiting inflammatory cells, and so, by amplifying VEGF and pro-inflammatory cytokine production [32].

Surprisingly, in our model, IL-1beta and TNF-alpha, which are among the more pro angiogenic inflammatory cytokines, were down regulated in the retina submitted to sustained PGF exposure. Indeed, PGF has been shown to trigger production of pro-inflammatory cytokines as TNF-alpha and IL-1beta in monocytes from patients with single cell disease [50], as well as TNF-alpha and IL-6 in the synovial tissue from patient with rheumatoid inflammation [51]. However, it has also been demonstrated that PGF modulates differentiation and maturation of dendritic cells in response to LPS, inhibiting NF-kappaB activity, and so inhibiting TNF-alpha production [52]. These studies suggest that PGF effects depend on target cells and on their environment. Our model showed that, after two months of production, rPGF-1 maintained VEGF expression promotion, but significantly down-regulated IL-1beta and TNF-alpha expression in retinal cells. This observation suggests that PGF may influence the cytokinic retinal micro-environment and may modulate the function of retinal inflammatory cells, as microglia. As IL-1beta and TNF-alpha are among the most pro-angiogenic inflammatory cytokines, their down-regulation may explain in part why no real vitreoretinal neovascularization occurred.

On the other hand, a significant increase in IL-6 resulted from PGF over expression. The exact significance of this increase remains to be determined, as IL-6 can have pro or anti inflammatory effects. Withal, a recent clinical study showed that ten weeks after intravitreal injection of bevacizumab (Avastin®, Genentech), a monoclonal antibody targeting VEGF, recurrence of edema occurred with low VEGF levels and high IL-6 levels in the aqueous humor [53], suggesting that PGF-induced IL-6 production may contribute to vascular permeability. The permeabilizing effect of PGF was also observed at the outer retinal barrier level, where disrupted occludin staining was observed at the tight-junctions together with changes in the morphology of RPE cells, suggesting alteration of this barrier, as previously described after acute PGF injection in the rat [19].

In the early phases of DR, alterations of retinal capillaries and focal aneurismal deformations progressively develop, very similar to what was observed in retina exposed chronically to PGF or in retina from GK rats. We therefore hypothesize that PGF could act in synergy with VEGF in the early phases of diabetic retinopathy. As dosing PGF/VEGF in the vitreous of patients at the early phases of the disease is not possible, we have looked at PGF expression in fibro-vascular membranes extracted from vitrectomy in patients with PDR. PGF was found not only in ECs but also in glial cells. We have also observed that PGF was expressed in glial cells in the normal rat retina and that PGF over-expression induced an intense glial activation. PGF could therefore also intervene in glial alterations observed in DR.

The implications PGF and its receptor has recently been demonstrated in several pathological conditions, particularly in oncology [32], leading to the development of new therapeutic molecules: TB-403 (Thrombogenics), a monoclonal anti-PGF antibody [54], and aflibercept (VEGF Trap®, Regeneron Pharmaceuticals/Bayer). This soluble VEGF receptor fusion protein designed to bind all forms of VEGF along with PGF, was formulated for intravitreal injections (VEGF Trap-Eye). These new drugs could have a therapeutic benefit for DR, and notably for diabetic macular edema. Positive results of a phase II study in patients with diabetic macular edema treated with VEGF Trap Eye came recently and showed a significant improvement in visual acuity over 24 weeks compared to macular laser therapy, demonstrating that blocking both Flt-1 ligands is efficient to limit macular edema formation [55]. Nevertheless, others GFs implied in DR, FGF-2 and EGF, may also be able to indirectly induce Flt-1 expression in ECs in a combinatorial fashion [56]. Thus, it would also be of interest to target Flt-1.

In summary, diabetic retinal changes might result from synergistic actions of several GFs. Our results suggest that PGF-1 could participate to vessel abnormalization observed in the early stages of DR. In patients, the vascular changes may result from the balance between VEGF/PGF and sFLT-1 in the ocular media at the different stages of the disease progression. Further studies are therefore required to analyze how the expression the VEGF receptors and their ligands are modulated during diabetic retinopathy.
